# Eye and Ear Clinic Atlanta Medical College

**Published:** 1875-01

**Authors:** L. K. Philpot

**Affiliations:** Reporter


### Clinic Atlanta Medical College.

L. K. PHILPOT, Reporter.

*A. W. Calhoun, M.D., Professor of Diseases of the Eye and Ear.*

Case 1, Nov. 6, 1874—Mary L., (white) æt. 4 years, Atlanta. We have in the patient before us a disease known as lymphatic or scrofulous ophthalmia, affecting both eyes and existing for two years. By examination, you will find some of the characteristic symptoms of this affection, viz: the narrow circle of ciliary blood vessels (ciliary injection) in the conjunctiva, around the periphery of the cornea, the little pimples upon the lower portion of the limbus corneæ, with minute vessels running into them from the conjunctiva, and several small opacities upon the cornea itself, indicating the previous existence of the disease, and now interfering somewhat with vision, as they are seated in front of the pupil. As is usual in such troubles, there is photophobia or intolerance of light, the child keeping its lids firmly closed when much exposed to light. The disease occurs mostly in scrofulous subjects, and is characterized by its frequent return, even after an apparently complete cure. Though well grown, this child is delicate. We will put the patient upon constitutional treatment, and prescribe as a local application to the eye the following:

℞.—Atropiæ Sulph., . . . . . gr. ij.

Aquæ Destil. . . . . . . . ℥ j.

M.S.—Drop few drops into the eyes several times daily. This will dilate the pupils, causing the blood vessels in the iris to contract and force much of the blood back from the front of the eye; and it will also relieve the morbid sensibility of the cornea. *Once daily* we will use *calomel*, by sprinkling a small quantity, with a camel's hair pencil, upon the mucous side of the lower lid, to produce absorption of the exudations upon the cornea. Preparations of iron and quinine will be given, and, if practicable, also cod liver oil.

To check an acute diarrhœa, the child will take the following:

℞.—Tinct. Krameriæ, . . . . . . .

Tinct. Opii. Camph., . . . . . aa ℥ j.

Cretæ Præcip., . . . . . . . . . ʒ ss.

M,S.—One-half to one teaspoonful every three or four hours.

Case 2, Nov. 6, 1874—S. J. H., (white) Rome, Ga., farmer, æt. 28 Years.—This is another case of scrofulous ophthalmia, and thus you have the opportunity of seeing the disease both in the child and the adult. In addition to the disease as you saw it in the last case, you have here a complication known as pannus. Pannus is not a distinct or separate disease, but secondary and always depending upon some previously existing trouble of the eye. It is a deposit upon the outer layer of the cornea, with vessels running into it from the corneal margin. As a rule, it begins at the periphery of the cornea, extending towards its centre with more or less rapidity, according to the severity of the disease, sometimes leaving the cornea so thickly covered and red as to resemble somewhat a piece of raw beef. Of course, the vision is defective in proportion to the thickness of the deposit and the vascularity, oftentimes completely blinding the patient. In this case it is only partial, and the result of the scrofulous disease, and will disappear as the affection does from which it originated.

For the same reasons as in the last case, the use of atropine is indicated (grs. ij. to ℥ j.) to dilate the pupil, obtund the morbid sensibility, and to act as a sedative to the inflamed eye. It is not in every case of scrofulous conjunctivitis that atropine is to be used—only in such as have the cornea seriously inflamed, or where the iris is in danger of becoming implicated. As a sedative and absorbent, the following salve is prescribed:

℞.—Ext. Belladonnæ, . . . . . . gr. xij.

Hyd. Præcip. Albi., . . . . . . gr. xij.

Ung. Simp., (or this: Ung.—Aquæ Rosæ,) . ʒ ij.

M. Ft. Ung.—S.—Rub a small piece above the eye-brow and the temple every two or three hours. (Note.—Patient recovered rapidly under the above.)

Case 3—Mrs. B., æt. 28, white, Chattanooga, Tenn.—We have here what was originally a typical case of purulent conjunctivitis or ophthalmia, resulting in a central corneal abscess and a perforating ulcer at the upper margin of the cornea. Under previous treatment, the abscess has very nearly disappeared, leaving a thick opacity. The bottom of the ulcer has ceased to secrete matter, and is now quite clean and healing. The ulcer did not literally perforate the cornea, but destroyed all the corneal layers down to the internal lining membrane (membrane of Descemet), which, being so thin, gave way under the pressure from the aqueous humour and bulged forward, looking like a small clear blister. As the ulcer healed, and under the pressure from the lid, this protrusion of the lining membrane has receded, you see now a clean, deep ulcer. As you have been told, the disease is seated in the mucous lining of the lids, and this will be treated by a solution of—

℞.—Argenti Nit., . . . . . . . gr. x.
Aquæ Destil. . . . . . . . . ℥ j.

M.S.—To be painted upon the inverted lids once or oftener, daily, according to the effect produced. Later, when the secretion of pus ceases, the lids will be bandaged, in order to prevent their rubbing over the ulcer and brushing away the new-formed epithelium. She can also use during the day, as a wash, a weak solution of zinc (gr. ij. to ℥ j.) This will hasten the cure. Opacities will remain where the ulcer and abscess were formed.

Case 4—R. P., colored girl, æt. 17 years, Atlanta.—In this girl you have a most dangerous disease, known as gonorrhœal ophthalmia. She has had gonorrhœa, and, for want of cleanliness, has carried some of the virus into the eye. The acute symptoms have passed away, fortunately, without affecting the other eye. As a consequence of this purulent inflammation, the papillæ in the conjunctiva of the upper lid have become enormously hypertrophied, and the cornea is one continuous vascular surface, constituting a complete pannus. Vision is, of course, entirely destroyed, and the deposit upon the cornea is so thick that we can never hope to remove it sufficiently to allow the entrance of light into the interior of the eye, to any great extent. The treatment must be mainly directed against the inflammation of the palpebral conjunctiva, which is the source of the whole trouble, and for this we prescribe—

℞.—Argenti Nit., . . . . . . . . gr. x.
Aquæ Destil.. . . . . . . ℥ j.

M.S.—To be painted upon the everted lids, by means of a camel's hair brush, once daily, or oftener, if necessary. In the interval between the pencilings she will use a solution of zinc (gr. ij—iv. to ℥ j.), to be dropped into the eye several times daily. Such inflammations, when becoming chronic, as in this case, are very stubborn, and the treatment must necessarily be continued for several months, requiring now and then a change in the treatment. It is best always to acquaint your patient with these facts.

CASE 5—J. L. G., CHARLOTTE, N. C., (WHITE,) ÆT. 25 YEARS.—In this man we have a case of pure granular lids—not hypertrophied papillæ, but a deposit of lymph underneath the conjunctiva, projecting forwards and forming true granulations. The disease has already existed six years, producing ulceration of the cornea, pannus, etc. From granular lids and scrofulous ophthalmia we have pannus occurring almost exclusively—at any rate, in the large majority of instances. The patient is almost completely blind, finding his way about with the greatest difficulty. Most cases of granular lids are favorable for a more or less complete cure, but require treatment continuing through several months. Nitrate of silver is the great remedy in such cases, using either the solid stick or the strong solution (gr. x. to ℥ j.) The solution can be applied more equally and advantageously than the stick. As long as pus is secreted, the nitrate of silver is indicated; when this ceases, then the solid stick of sulphate copper, painted daily over the conjunctiva, acts beneficially. The two can be used alternately. As the granulations disappear, so will the pannus and the ulcers. Under this treatment the patient has, in a short time, improved very rapidly.

CASE 6—T. B., ATLANTA, (COL'D,) ÆT. 12 YEARS.—Another typical case of scrofulous ophthalmia, in which the constitutional taint is clearly marked. We often have the disease without any clearly defined constitutional symptoms, but here they are prominent. The cervical glands are much swollen; there is an eczematous inflammation around the entrance of the nose; and, besides, the general appearance of the boy would lead you to this conclusion. Aside from the general symptoms, the inflamed eye itself has every appearance of the scrofulous inflammation which were described to you in a previous case. For the same reasons as before mentioned, a solution of atropine (gr. ij. to ℥ j.) will be dropped into the eyes several times daily, and once daily calomel will be sprinkled upon the inside of the lower lid—or, instead of this, use a salve, viz:

℞.—Hyd. Ox. Flav., . . . . . . gr. iij.
Ung. Simp. (or Ung. Aquæ Rosæ), . . ℥ ij.

M.S.—Place a small piece upon the inside of lower lid, every night, upon going to bed.

By watching this case, you will see the tendency of the disease to return, for he has been apparently cured several times, when, upon slight exposure, the affection would reappear. He is now rapidly improving and growing stronger, but his general health must be attended to. To relieve the photophobia, the *compound belladonna ointment* can be advantageously used, as before described.

CASE 7—COLORED GIRL, ÆT. 16 YEARS, ATLANTA.—For the removal of a total staphyloma of the left eye, this girl presents herself. From long continued inflammation, the cornea became diseased and softened, and the contents of the eye have forced the diseased tissues forward, until the protrusion extends out between the lids, constituting total staphyloma. Vision is gone, and the only thing to be done is to remove the deformity. Patient is chloroformed, and the lids held apart by means of the eye speculum, and Critchett's operation made upon the staphyloma. Five large curved and threaded needles are passed entirely through the ball, immediately behind the enlargement, coming through below at a point corresponding to their entrance above. All the projecting portion of the eye in front of the needles (leaving only sufficient room for the needles to hold) is removed by means of the knife and scissors. The needles and threads are then carefully drawn through, and each firmly tied, and the eye bandaged. Nothing but cold water dressings were used, the sutures being allowed to remain one week, when, upon removal, the cut edges were firmly grown together. Patient suffered no very severe pain during the healing process. All inflammation rapidly subsided, and to-day (three weeks after operation) the patient is brought before you to show you the complete removal of the staphyloma, and the manner of fitting on a glass eye. The artificial eye goes in easily, and adjusts itself readily to the stump of the globe. Indeed, it fits so well, and has all the natural movements of a sound eye so perfectly, and corresponds so nearly in color to the other, that it is almost impossible to tell which is the natural or which is the artificial eye. The wearing of it is a real comfort to her, and the only caution necessary is to remove it each night and put it into a glass of water, and replace it in the morning.

CASE 8—C. C., COLORED, ÆT. 58 YEARS.—To-day we shall make an operation for the removal of cataract in this man, who comes to us from Florida. You have had explained to you that cataract is a disease of the crystalline lens and its various characteristics. It is mature in the left eye, and immature or *unripe* in the right; hence we shall operate only upon the one eye, the left. As usual, I shall use no chloroform in this operation, wishing always to have the assistance of the patient. *Graefe's linear extraction* is best suited to such cases. The manner in which it is made, you have had explained to you, and have seen it also several times. The cut must be made through the cornea, then the iridectomy and the rupture of the anterior capsule of the lens, and then the removal of the lens itself. Three weeks after the first operation, the patient is brought before you again to clip off the prolapsed portion of the iris, which escaped through the corneal wound upon the patient making a straining effort. The prolapsus has also drawn the pupil so high up that it is covered by the upper lid; and at the same time that I remove the prolapsed portion of the iris, I shall also make an *iridotomy*, and thereby extend the pupil downwards from beneath the upper lid. To-day (two weeks after the second operation) you see the prolapsus gone, and a broad slit (artificial pupil) in the iris. Patient has good vision, being enabled to go everywhere by himself, and will return home in a few days. His exact vision has not yet been tested, but will be in a few days, when cataract glasses will also be adjusted to the eye. Note.—With No. 4 cataract glass vision is two-thirds; with No. 2½ cataract glass he reads small type.

CASE 9—M. F., COLORED, ÆT. 60 YEARS, FROM MADISON, GA.—Another case of mature cataract in both eyes. The same operation (Graefe's linear extraction) will be made as in the last case of cataract, making one operation at a time—to-day upon the right eye. Two weeks after the first operation, the patient is again brought to the Clinic for the removal of the cataract from the left eye, the right eye having entirely healed, and his vision being as perfect as possible, as you perceive he is able, with the aid of a glass, to tell the time of day by the watch. The same operation is here also made, and the same after treatment gone through with. Three weeks after the second operation (five weeks since first operation), we bring the patient before you to show the complete success upon both eyes. The inflammation has completely subsided in the right, and very nearly in the left eye; and after testing exactly his vision, and adjusting cataract glasses, the old man will be sent home as a perfect cure. As you see, he has no trouble, even without glasses, in going where he wishes and attending to whatever he may have to do.

